# P53 and pRB induction improves response to radiation therapy in HPV-positive laryngeal squamous cell carcinoma

**DOI:** 10.1016/j.clinsp.2024.100415

**Published:** 2024-06-18

**Authors:** Weiquan Ding, Weiwei Cai, Haili Wang

**Affiliations:** Department of Otolaryngology Head and Neck Surgery, Panyu Central Hospital, Guangzhou, Guangdong, China

**Keywords:** LSCC, Radiation, p53, RB, HPV

## Abstract

**Introduction:**

Patients with Human Papillomavirus (HPV+)-associated Laryngeal Squamous Cell Carcinoma (LSCC) exhibit dramatically improved survival relative to those with HPV-Negative (HPV-) tumors. In this study, the authors aimed to investigate the radiosensitivity of all available confirmed HPV+ and HPV-LSCC cells *in vitro* and *in vivo*.

**Methods:**

Primary LSCC cells were generated from tumor specimens obtained from patients. Real-time PCR was performed to confirm HPV infection and the expression of HPV-related genes (E6 and E7), p53, and pRB. Clonogenic survival assays, western blotting, and flow cytometry were used to assess radiation sensitivity, apoptosis, and the expression of p53 and pRB. p53 and pRB knockout cells were generated using CRISPR/Cas9 technology.

**Results:**

HPV+ LSCC cells displayed enhanced radiation sensitivity compared to HPV- cells. Radiation-induced apoptosis in HPV+ LSCC cells, accompanied by increased levels of p53 and pRB. Knockout of p53 or pRB led to radiation resistance and attenuated radiation-induced apoptosis in HPV+ LSCC cells. In vivo experiments showed similar results, where knockout of p53 or pRB decreased radiosensitivity in tumor-bearing mice.

**Conclusion:**

The present findings demonstrated that HPV+ LSCC cells displayed obvious inherent radiation sensitivity, corresponding to increased apoptosis following radiation exposure. Mechanism study showed that the expression of p53 and pRB in HPV+ cells are required for radiation sensitivity. These findings highlight a novel mechanism by which p53 and pRB play key roles in the radiation sensitivity of HPV+ LSCC compared to HPV-LSCC.

## Highlights

• Apoptosis is required for radiation sensitivity in HPV+ LSCC cells.
• P53 and pRB are required for radiation sensitivity of HPV+ LSCC cells.
• P53 and pRB are required for radiation sensitivity of HPV+ LSCC tumors *in vivo*.

## Introduction

Laryngeal cancer is a head and neck tumor that has a high incidence.^1^ In addition, Laryngeal Squamous Cell Carcinoma (LSCC) is the most prevalent pathological type of laryngeal cancer.^1^^,^^2^ Early LSCC can be treated using surgery or radical radiotherapy, whereas late LSCC requires comprehensive treatment involving chemotherapy, radiation, and surgery together with multidisciplinary management of toxicity and follow-up regimens.^3^^,^^4^ Thus, it is necessary to treat LSCC to ameliorate the effects of radiation. Although radiation technology has greatly improved recently, the survival effect on LSCC remains unexplored because of radioresistance.^5^^,^^6^ For these patients, the radiation dose may need to be increased, accompanied by a higher risk of impairing critical surrounding organs, causing more toxic effects such as dysphagia.

Many studies have confirmed that HPV mainly combines with the tumor suppressors p53 and pRB through the E6 and E7 proteins to cause uncontrolled cell proliferation, resulting in damage or even loss of cellular DNA self-repair function, leading to cancer.^7^, ^8^, ^9^, ^10^ The characteristic of HPV E6 protein is that it has two structural domains, which contain zinc finger structures, and these zinc finger structures bind to DNA and RNA and mediate protein-protein interactions to realize the cell transformation of E6 protein.^11^ The E6 protein mainly causes mutations in the tumor suppressor gene p53 and binds to the p53 protein, leading to its inactivation and degradation of the p53 protein, thereby causing malignant expansion of cells.^12^^,^^13^ However, during radiotherapy, p53 may be partially activated, thereby inducing cell death.^14^ In addition, p53 knockdown increased radiotherapy resistance, which further supports the possible role of p53 in radiotherapy. pRB is an RB gene-encoded protein that regulates the entry of cells into the S phase by binding to the E2F family of transcription factors.^15^ In normal cells, pRB is phosphorylated by the CDK4/CDK6/cyclin D complex during the G1 phase.^16^ Phosphorylated pRB decomposes the pRB/E2F transcriptional repression complex, releases E2F, and activates genes that enter the S phase for cell division.^16^^,^^17^ On the one hand, HPV E7 protein can weaken the inhibitory effect on the E2F transcription factor by promoting the phosphorylation of Prb.^18^^,^^19^ Nevertheless, there is currently a lack of relevant research on whether the function of HPV-positive pRB changes under radiotherapy and affects radiotherapy sensitivity.^20^

Although p53 and pRB play important regulatory roles in HPV-positive tumors, research on the radiosensitivity of p53 and pRB in LSCC is lacking. Therefore, in this study, the authors investigated the *in vivo* and *in vitro* effects of radiotherapy on LSCC with different expressions of HPV (+/-) and observed its effect on p53 and pRB expression to investigate their effect on radiotherapy sensitivity and provide a basis for clinical radiotherapy.

## Materials and methods

### Cells generation and culture

This study has been approved by the Ethics Committee of Panyu Central Hospital (protocol number PYRC-2021-060). The Animal and Clinical Study should follow the ARRIVE guidelines. All study participants provided written informed consent before participating in the study. Fresh tumor specimens were obtained from LSCC patients, who went through surgery, and instantly immersed in RPMI 1640 medium added amphotericin B (0.25 μg/mL) as well as penicillin/streptomycin (100 U/mL/0.1 mg/mL). The specimens were rinsed three times with PBS and subsequently cut into small pieces. Next, small tumor masses were enzymatically dissociated at 37°C in RPMI 1640 medium added type IV collagenase (200 U/mL) for 12 hours. After washed twice with PBS and centrifuging, the precipitate was inoculated onto Petri dishes (60 mm) and cultured in a complete epithelial cell medium supplemented with 1 % penicillin/streptomycin. After incubation for 3 days, the medium was changed. Cells were passaged every 3–4 days. Trypsin digestion (0.25 % trypsin-EDTA) was performed to clear cancer-associated fibroblasts (CAFs).

### HPV validation by Real-time PCR

Real-time PCR was used to confirm HPV-16 E6, and E7 transcription on a Bio-Rad CFX96 using primers and probes provided by Integrated DNA Technologies. In brief, total RNA was collected from both HPV+ and HPV- cells through miRNeasy with RNeasy MinElute Cleanup Kit (Qiagen). cDNA was synthesized using the iScript Reverse Transcription Supermix Kit (Bio-Rad Laboratories). Real-time PCR was performed with 10 ng of cDNA per 10 mL of reaction volume. The transcripts of GAPDH, E6, E7, p53, and pRB were examined. The thermocycler was set first at 95°C for 3 min, then 35 cycles at 94°C for 15s and 60°C for 0.5 min. Primers are list as follows: GAPDH: Forward: 5’-GGACCTGACCTGCCGTCT-3’, Reverse: 5’-TAGCCCAGGATGCCCTTG-3’; E6: Forward: 5’-AATGTTTCAGGACCCA-3’, Reverse: 5’-GTTGCTTGCAG-TACACACAT-3’; E7: Forward: 5’-GAGGAGGAGGATGAAA-3’, Reverse: 5’-GCACAACCGAAGCGTA-3’; p53: Forward: 5’-TCAGCATCTTATCCGAGT-3’, Reverse: 5’-TGGATGGTGGTACAGTCA-3’; pRB: Forward: 5’-TCAGCCAGGAAGAATCTCCC-3’, Reverse: 5’-GAGGTTTGTTGCCTCC-TTGT-3’.

### Clonogenic survival assay

Cell clonal survival after radiation was assessed as previously described using a JL Shepherd ^137^Cs irradiator at a dose delivery rate of 400 cGy/min.^21^ After 10–15 days, colonies with more than 50 cells were counted, the survival proportion was obtained, and the clonogenic survival curves conformed to a linear-quadratic model.

### Western blotting

After treatment, the cells were lysed using RIPA buffer. Protein (50 μg) was analyzed via SDS-PAGE and subsequently added onto PVDF membranes, which were observed using specific primary antibodies through incubation with secondary antibodies conjugated to horseradish peroxidase. The signal was visualized with an enhanced chemiluminescent substrate (Thermo Scientific).

### Apoptosis detection

Apoptosis was determined using Flow Cytometry (FC) by examining the changed lipophilic-packing plasma membrane phospholipid, Annexin V conjugated to FITC (BD Biosciences) based on the instruction. Early apoptosis (Annexin V-positive, propidium iodide-negative), late apoptosis (both positive), and live (both negative) cells were examined using FACSCalibur FC and investigated using FlowJo v9.4.3.

### P53 and pRB knockout cells generation

The sgRNAs targeting p53 and pRB were designed using CRISPR and synthesized by Sangon Biotech Co., Ltd. (Shanghai, China). Oligonucleotides were cloned into the Cas9 backbone Lenti-CRISPR v2 vector. HEK293T cells were co-transfected with recombinant plasmids psPAX2 and pVSVG. The medium was collected and filtered after 48 h. Following infection, cells were obtained through cultivation with 4 g/mL puromycin for more than 14 days. Single clones were selected, and western blotting was used to identify p53 or pRB knockouts.

### Xenografts tumor experiments

Female nude mice (28- to 35-day old) were provided by Harlan Laboratories, kept in filter-topped cages under aseptic conditions, and maintained according to a protocol approved by the Committee of Panyu Central Hospital. The bilateral flanks were injected (s.c.) with cells (1–2 × 10^6^ mixed with Matrigel, 1:1) for xenograft establishment. When the tumor reached approximately 100 mm^3^, the mice (n = 12/group) were arbitrarily exposed to 4 Gy delivered in four 2 Gy fractions or mock radiation for more than 10 days. Radiation was delivered at a dose rate of 0.6 Gy/min or so via an X-Rad 320 biological irradiator. Specific lead immobilization jigs were used to protect most of the mouse body during tumor exposure. Tumor size was determined, and a growth curve was obtained. All procedures were approved by the Animal Care and Use Committee of Panyu Central Hospital.

### Statistical analysis

Data were analyzed using GraphPad Prism VI. Student's *t*-test was used to detect differences between two experimental groups. Pearson's correlation analysis was used to analyze the connections among genes. The survival rate was determined using the Kaplan-Meier method; p < 0.05 meant statistical significance.

## Results

### LSCC cells generation

**Fig. 1:**
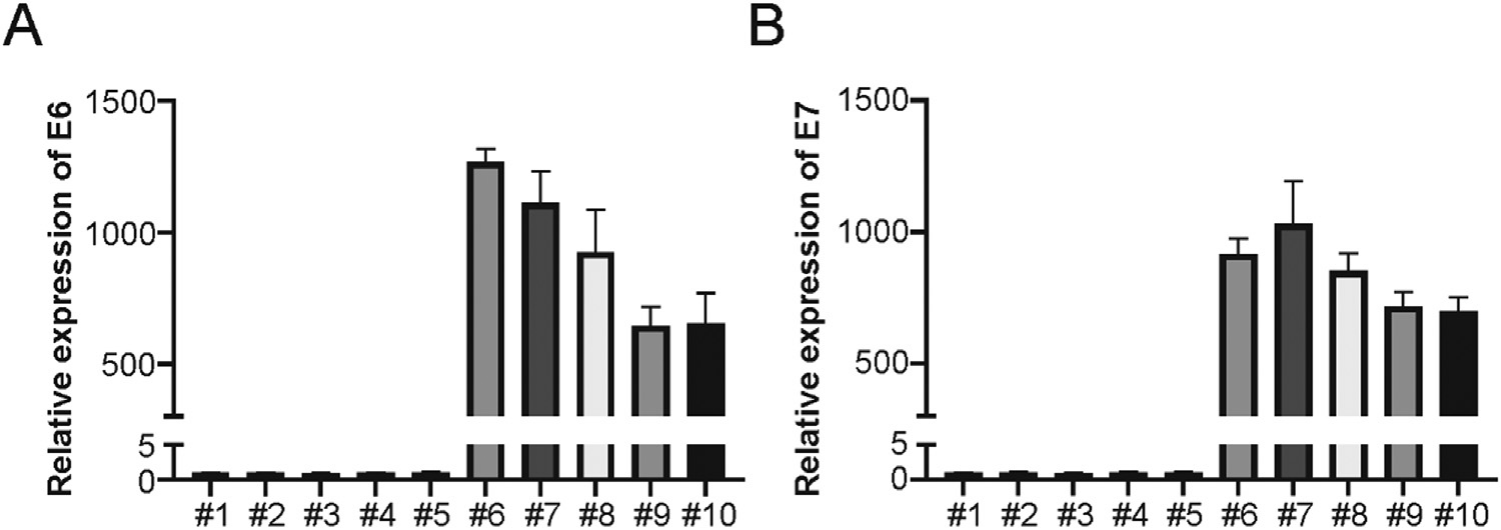
Generation of LSCC cells and HPV status identification. (A) HPV E6 was analyzed by Real-time PCR in primary LSCC cells. (B) HPV E7 was analyzed by Real-time PCR in primary LSCC cells.

To investigate the role of HPV infection in the radiation sensitivity of LSCC cells, the authors generated a series of primary LSCC cells and identified HPV infection. Real-time PCR was utilized for confirming HPV+ and HPV- cells, with or without HPV16 E6 and E7 gene expression, respectively. The authors generated 5 HPV+ and 5 HPV- LSCC cell lines (Fig. 1A and B). The present results suggest that HPV+ and HPV- LSCC cells were successfully generated.

### Enhanced sensitivity of HPV+ LSCC to radiation

**Fig. 2:**
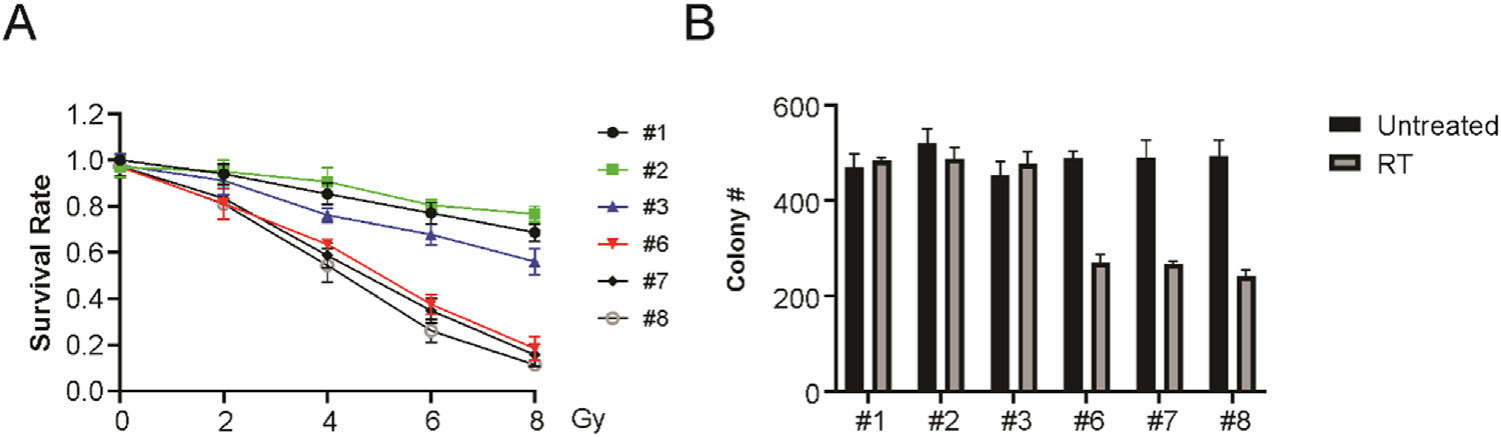
HPV+ LSCC cells are more sensitivity to radiation. (A) Clonogenic survival of selected HPV- and HPV+ LSCC cells post radiation treatment (4 Gy) as indicated. (B) Colony formation assay of indicated HPV- and HPV+ LSCC cells post radiation for 14 days.

A panel of six LSCC cell lines, including 3 HPV+ and 3 HPV- LSCC cell lines, were used to probe radiation sensitivity via a clonogenic survival assay. These findings indicated that HPV+ LSCC cells exhibited significantly greater radiation sensitivity (Fig. 2A and B). Therefore, the above results suggest that HPV+ LSCC cells are more sensitive to radiation than HPV-LSCC cells.

### Apoptosis induction in HPV+ LSCC upon radiation

**Fig. 3:**
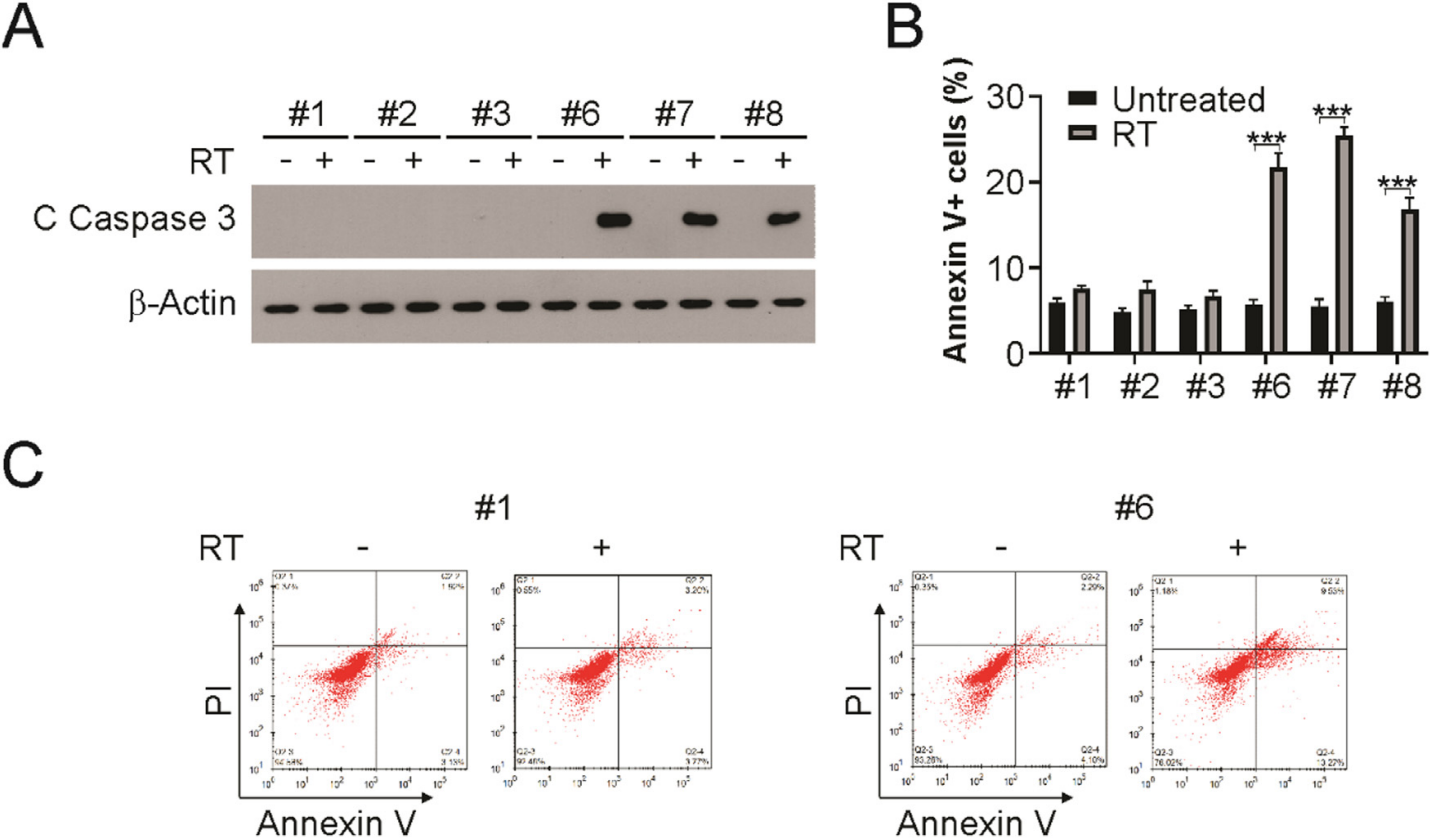
Apoptosis induction is required for radiation sensitivity in HPV+ cells. (A) Indicated cells were treated with 4 Gy radiation, 24 hours later, cleaved caspase 3 was detected by western blotting. (B and C) Indicated cells were treated with 4 Gy radiation, 24 h later, apoptosis was analyzed using flow cytometry. The results in (B) are presented as Means ± SD (*** *p* < 0.001).

Patients with HPV+ LSCC exhibited an earlier tumor response than those with HPV- LSCC. Radiation sensitivity is related to apoptotic tumor responses in other cancer types. Therefore, the authors analyzed the apoptosis induction post-radiation in HPV+ and HPV- cell lines. As shown in Fig. 3A, radiation treatment induced caspase 3 activation in HPV+ LSCC cells, but not in HPV- cells. In addition, Annexin V/PI assay was used to evaluate apoptosis in radiation-exposed HPV+ and HPV- cells. HPV- cells displayed only minor elevation in apoptosis, while HPV+ cells displayed strong apoptosis induction (Fig. 3B and C). Therefore, the above findings demonstrate that apoptosis is required for radiation sensitivity in HPV+ LSCC cells.

### Induction of p53 and pRB in HPV+ LSCC cells upon radiation

**Fig. 4:**
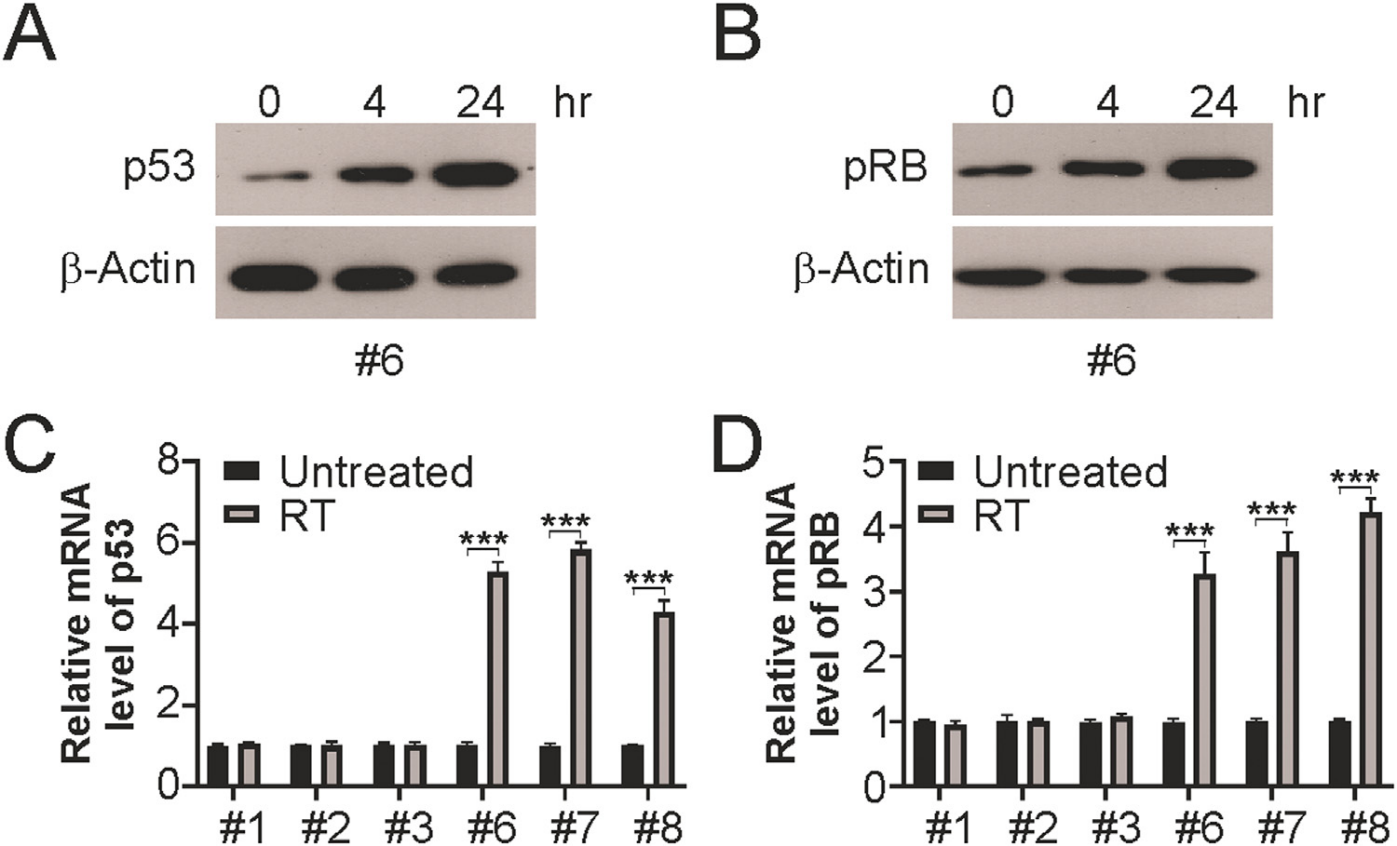
p53 and pRB are induced by radiation in HPV+ cells. (A) Indicated cells were treated with 4 Gy radiation, 24 hours later, p53 protein level in irradiated HPV+ cells were analyzed by western blotting. (B) Indicated cells were treated with 4 Gy radiation, 24 hours later, pRB protein level in irradiated HPV+ cells were analyzed by western blotting. (C) Indicated cells were treated with 4 Gy radiation, 24 hours later, p53 mRNA level in indicated cells were analyzed by Real-time PCR. (D) Indicated cells were treated with 4 Gy radiation, 24 hours later, pRB mRNA levels in indicated cells were analyzed by Real-time PCR. The results in (C and D) are presented as Means ± SD (***p < 0.001).

To determine the functional role of p53 and pRB in the radiation sensitivity of LSCC cells, p53 and pRB levels were evaluated at 4h and 24h following radiation. Elevated levels of total p53 and pRB were observed at the indicated time points after irradiation (Fig. 4A and B). In line with the induction of p53 and pRB by radiation, the mRNA levels of p53 and pRB were also increased in HPV+ LSCC cells, but not in HPV-LSCC cells (Fig. 4C and D). These findings suggested the presence of p53 and pRB in HPV+ cells after radiation treatment.

### Knockout of p53 or pRB leading to radiation resistance

**Fig. 5:**
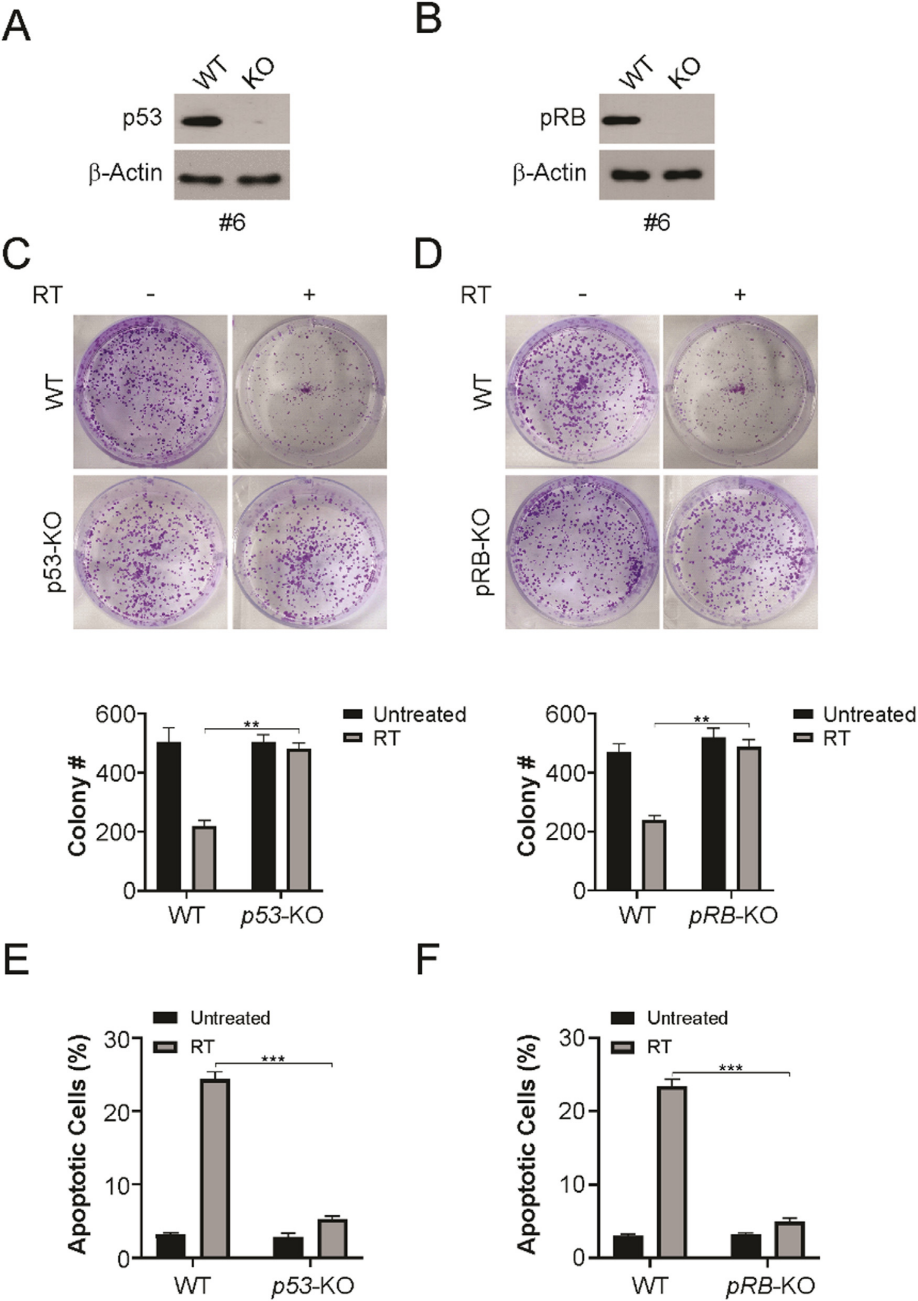
p53 and pRB knockout HPV+ LSCC cells resistance to radiation. (A) p53 knockout HPV+ cells (#6) were identified by western blotting. (B) pRB knockout HPV+ cells (#6) were identified by western blotting. (C) WT and p53-KO HPV+ cells were treated with 4 Gy radiation, 14 days later, colony formation assay was performed. (D) WT and pRB-KO HPV+ cells were treated with 4 Gy radiation, 14 days later, colony formation assay was performed. (E) WT and p53-KO HPV+ cells were treated with 4 Gy radiation, 24 hours later, cleaved caspase 3 was detected by western blotting. Apoptosis was analyzed by counting cells with condensed and fragmented nuclei. (F) WT and pRB-KO HPV+ cells were treated with 4 Gy radiation, 24 hours later, cleaved caspase 3 was detected by western blotting. Apoptosis was analyzed by counting cells with condensed and fragmented nuclei. The results in (C‒F) are presented as Means ± SD (** *p* < 0.01; *** *p* < 0.001).

To determine the requirement of p53 and pRB in radiation sensitivity, p53 or pRB were knocked out in HPV+ LSCC cells using sgRNAs specific for p53 or pRB (Fig. 5A and B). Using clonogenic survival assays, radiation decreased the survival rate in WT HPV+ LSCC cells. However, p53 or pRB knockout caused an obvious increase in colony formation, in line with radiation resistance in HPV+ LSCC cells (Fig. 5C and D). In addition, apoptotic cell analysis indicated that knocked out of p53 or pRB attenuated radiation-induced apoptosis in HPV+ LSCC cells (Fig. 5E and F). The present findings indicate that p53 and pRB are required for the radiation sensitivity of HPV+ LSCC cells.

### P53 and pRB regulate radiation sensitivity in vivo

**Fig. 6:**
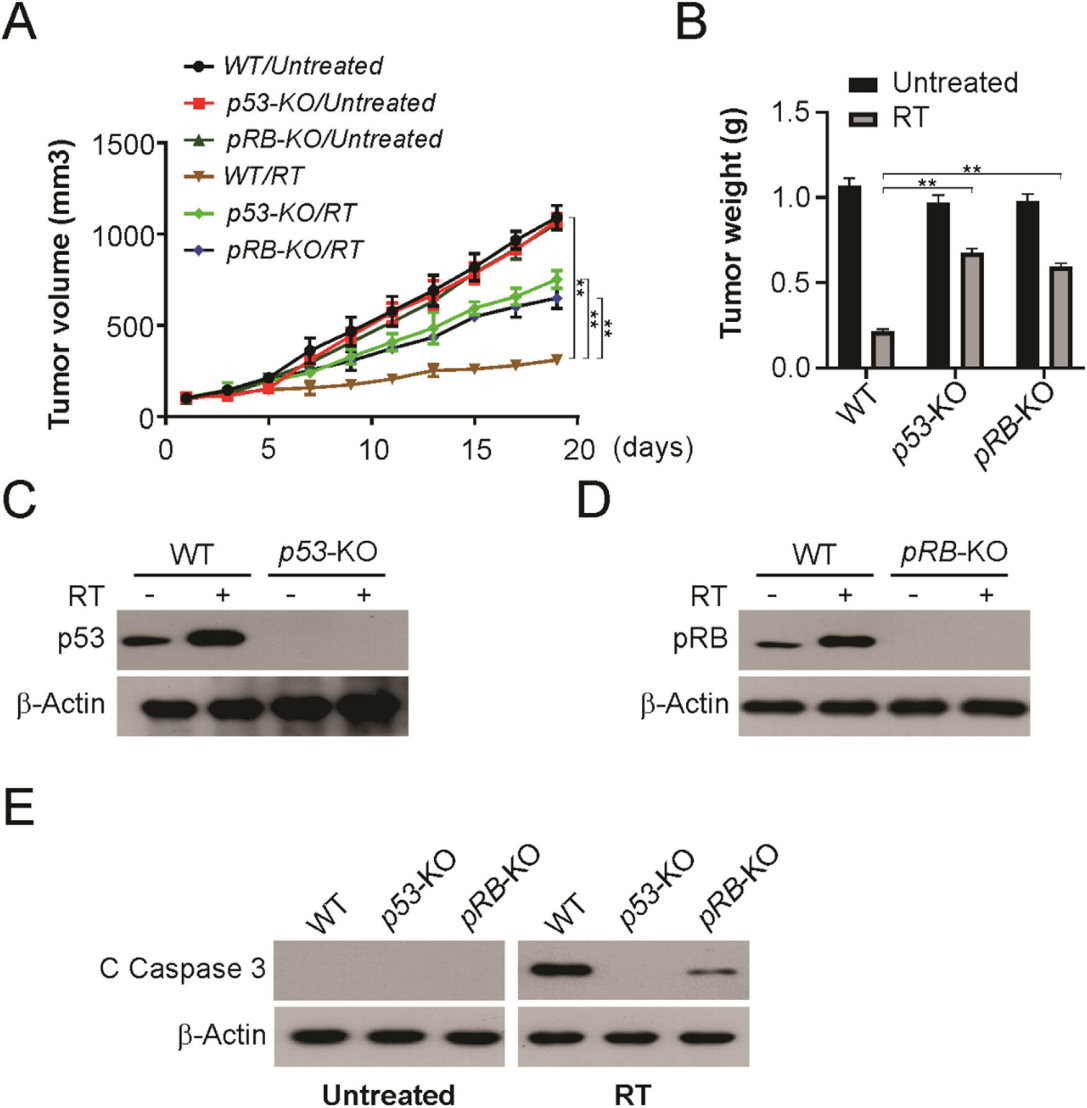
Both of p53 and pRB are required for radiation therapy *in vivo*. (A) Tumor volume curves of WT, p53-KO and pRB-KO xenograft with or without irradiation treatment (4 fractions of 2 Gy). (B) Tumor weight of WT, p53-KO and pRB-KO xenograft with or without irradiation treatment at indicated in (A). (C) p53 level of WT and p53-KO xenograft with or without irradiation treatment at indicated in (A). (D) pRB level of WT and pRB-KO xenograft with or without irradiation treatment at indicated in (A). (E) Cleaved caspase 3 of WT, p53-KO and pRB-KO xenograft with or without irradiation treatment at indicated in (A) was detected by western blotting. The results in (B) are presented as Means ± SD (** *p* < 0.01).

To confirm whether the enhanced radiosensitivity observed *in vitro* could be detected *in vivo*, the authors established tumors using WT, p53-KO, and pRB-KO HPV+ LSCC cells. Knockout of p53 or pRB significantly decreased radiosensitivity compared to WT HPV+ LSCC cells (Fig. 6A and B). p53 or pRB induction was absent in knock-out tumors (Fig. 6C and D). Furthermore, knockout p53 or pRB blocked radiation-induced apoptosis in tumors (Fig. 6E). Therefore, these findings demonstrate that p53 and pRB are required for radiation sensitivity of HPV+ LSCC tumors *in vivo*.

## Discussion

Laryngeal squamous cell carcinoma currently ranks second in terms of the incidence of malignant tumors of the head and neck.^22^ Currently, domestic clinical treatment focuses on the surgical treatment of early- and mid-stage laryngeal cancer.^23^ Radiotherapy can preserve the laryngeal function. However, it has been clinically observed that some laryngeal cancer patients are less sensitive to radiotherapy, and they are prone to early recurrence after radiotherapy.^24^ If radiotherapy is chosen instead of surgery for these patients, they are at risk of reoperation after radiotherapy failure.^24^ Therefore, it is necessary to accurately screen patients sensitive to radiotherapy, so as to select the most suitable treatment method.

Human Papillomavirus (HPV) infection leads to squamous cancers of the oropharynx, and more studies suggested that HPV infection is also relative to an increased risk of Laryngeal Squamous Cell Carcinoma (LSCC).^25^ There is increasing evidence showing that LSCC is associated with HPV infection, but the role of HPV in LSCC has not been conclusively established.^25^^,^^26^ Recent studies have shown that HPV infection is not only tightly associated with laryngeal cancer occurrence and progression but is also related to the sensitivity of oropharyngeal tumors to radiotherapy.^27^ Currently, the influence of HPV infection on the radiosensitivity of oropharyngeal tumors is controversial, and most studies have suggested that HPV-positive oropharyngeal tumors can increase radiosensitivity.^28^ However, other studies have suggested that HPV-positive oropharyngeal tumors not only fail to increase radiosensitivity but also reduce radiosensitivity.^29^ At present, it is unknown whether HPV-positive LSCC affects radiotherapy sensitivity, and there is a lack of relevant research on radiotherapy sensitivity of LSCC cell lines, and there is also a lack of corresponding research on whether HPV infection affects laryngeal cancer radiotherapy sensitivity.^30^, ^31^, ^32^ An in-depth understanding of the possible role of HPV in enhancing radiosensitivity in LSCC and its mechanism, as well as locating and identifying reliable biomarkers of sensitivity, has important clinical significance for guiding treatment and has become urgent work.

In the current study, the authors investigated the effect of radiotherapy on HPV-positive/negative LSCC cell lines and the *in vivo* effect in nude mouse tumor-bearing models, as well as the activity and expression of the HPV-related proteins p53 and pRB, to determine whether HPV infection affects radiotherapy sensitivity. Firstly, the authors used real-time PCR to identify the HPV infection in primary LSCC cells and found that 5 HPV+ and 5 HPV- LSCC cell lines were generated successfully. Cell survival assay, colony formation, western blot, and flow cytometry were used to detect the sensitivity and apoptosis of these cells to radiation therapy. Furthermore, the authors used CRISPR/Cas9 system to knocked-out p53 and pRB in HPV+ LSCC cells and found both p53 and pRB can affect the sensitivity to radiotherapy and the specific molecular mechanism of action and provide a more sufficient theoretical and experimental basis for the selection of patients with laryngeal cancer radiotherapy advantages. Both HPV+ and HPV- cells displayed significant variations in radiation sensitivity, which is definitely true in the clinical domain, where patients with LSCC show a wide radiation response spectrum; thus, the authors expected a wide response heterogeneity in the preclinical setting. However, these systematic assessment of HPV+ LSCC cells showed an obvious pattern of elevated radiosensitivity relative to that of HPV- LSCC cells. Radiation sensitivity appears to be related to radiation-induced apoptosis in HPV+ LSCC cells.

The mechanism of the anticancer effect of radiation is a complex network of interconnected signaling pathways that leads to apoptosis, cell cycle arrest, and DNA repair.^33^ Various factors, such as p53, PARP, DNA-PK, and ATM, have been reported to be associated with radiation-induced DNA damage and apoptosis.^34^ p53 plays a key role in the cellular response to radiation by modulating the transcriptional activation of many downstream targeting genes, including GADD45, p21, Bax,14-3-3, and Mdm2.^35^^,^^36^ Expression of Wild-Type (WT) p53 demonstrates higher radiation sensitivity than the defective type.^35^ Furthermore, radiation-sensitive tissues usually have a higher basic p53 mRNA expression and are more susceptible to apoptosis-inducing responses to radiation exposure, suggesting that radiation can enhance cell apoptosis in p53-dependent pathways.^34^ Following radiation-induced DNA damage, the p53 pathway could be activated to repair DNA.^34^ However, this damage is not limited in DNA repair, p53 directly induces PUMA, Bax and Bcl-2 by activating transcription of apoptotic genes, thus regulating the occurrence of apoptosis in cancer cells.^37^ In addition, the role of the pRb protein in the cell cycle and apoptosis interface decision-making has been determined. Loss of pRb function leads to increased apoptosis, primarily due to the release of free E2F cell cycle regulators.^38^ Hypophosphorylated pRb also triggers apoptosis, which is then cleaved by caspases.^39^ In the current study, the present findings indicated that radiation-induced apoptosis in a p53- and pRB-dependent manner in HPV+ LSCC.

In short, the study indicated that relative to HPV-LSCC cells, those originating from HPV+ LSCC exhibited elevated inherent radiation sensitivity, suggesting a correlation between p53 and pRB induction and a strong apoptotic response.

## Funding

This research was supported by Science and Technology Projects in Guangzhou (grant no. 202102080536).

## CRediT authorship contribution statement

**Weiquan Ding:** Conceptualization, Data curation, Methodology, Writing – original draft, Writing – review & editing. **Weiwei Cai:** Conceptualization, Formal analysis, Methodology, Resources. **Haili Wang:** Formal analysis, Investigation, Project administration.

## Declaration of competing interest

The authors declare no conflicts of interest.
